# Restricted access: spatial sequestration of damaged proteins during stress and aging

**DOI:** 10.15252/embr.201643458

**Published:** 2017-02-13

**Authors:** Sandra Malmgren Hill, Sarah Hanzén, Thomas Nyström

**Affiliations:** ^1^ Institute of BiomedicineSahlgrenska AcademyUniversity of GothenburgGöteborgSweden

**Keywords:** aging, asymmetric division, protein aggregates, protein quality control, vesicle trafficking, spatial quality control, Ageing, Protein Biosynthesis & Quality Control

## Abstract

The accumulation of damaged and aggregated proteins is a hallmark of aging and increased proteotoxic stress. To limit the toxicity of damaged and aggregated proteins and to ensure that the damage is not inherited by succeeding cell generations, a system of spatial quality control operates to sequester damaged/aggregated proteins into inclusions at specific protective sites. Such spatial sequestration and asymmetric segregation of damaged proteins have emerged as key processes required for cellular rejuvenation. In this review, we summarize findings on the nature of the different quality control sites identified in yeast, on genetic determinants required for spatial quality control, and on how aggregates are recognized depending on the stress generating them. We also briefly compare the yeast system to spatial quality control in other organisms. The data accumulated demonstrate that spatial quality control involves factors beyond the canonical quality control factors, such as chaperones and proteases, and opens up new venues in approaching how proteotoxicity might be mitigated, or delayed, upon aging.

**Glossary**
AGGs: asymmetry‐generating genes
ALS: amyotrophic lateral sclerosis
CCT: chaperonin containing TCP‐1
CVT: cytoplasm‐to‐vacuole targeting
ER: endoplasmic reticulum
INQ: intranuclear quality control
IPOD: insoluble protein deposit
JUNQ: juxtanuclear quality control
PAS: pre‐autophagosomal structure
PQC: protein quality control
Prx: peroxiredoxin
ROS: reactive oxygen species
RQC: ribosome quality control
sHsps: small heat shock proteins
UPS: ubiquitin proteasome system
VHL: von Hippel–Lindau tumor suppressor

## Introduction

Proteins are responsible for cellular structure, function, and regulation. The maintenance of protein homeostasis, or proteostasis, is therefore crucial for survival, and cells have evolved an intricate system of temporal protein quality control (PQC) that ensures proper production, renewal, and function of the proteome 1, 2, 3. The chaperones of the temporal PQC system govern the *de novo* folding of nascent polypeptides and the refolding of proteins that have lost their tertiary structure. Chaperones also promote, together with proteases, the degradation of proteins that cannot be refolded 4, 5. Removing misfolded proteins from the cell is a crucial process, and if this fails, misfolded proteins can accumulate in differently sized aggregates and larger inclusions 6, 7. Such aggregated proteins (or oligomers on their path to aggregates) are harmful, not only due to the loss of function of the non‐native proteins, but perhaps mostly due to toxic gain of function through interaction with, and impeding of, essential processes 8, 9. The importance of proteostasis is exemplified by the many diseases that are associated with protein misfolding and aggregation such as cystic fibrosis, amyotrophic lateral sclerosis (ALS), Alzheimer's and Parkinson's disease, where proteins of aberrant structure interfere with cellular function and eventually cause cell death 3, 10, 11.

The cellular PQC system is continuously at work to maintain low levels of protein damage; however, the system is imperfect in the sense that it is tuned for optimal reproduction success and not for long‐term survival 12, 13. Thus, accumulation of protein damage appears inevitable during aging. To cope with the accumulation of damaged and aggregated proteins, cells have, in addition to temporal PQC, developed a second line of defense where oligomers and aggregated proteins are sequestered into large inclusions at certain protective locations within the cell 14, 15, 16, 17. This process of spatial PQC harnesses the toxicity of misfolded proteins, oligomers, and aggregates, and can aid in their subsequent clearance 18, 19, 20. Spatial sequestration also facilitates the asymmetric segregation of damaged proteins that occurs during cell division, which enables an old progenitor cell to produce a pristine daughter cell harboring a “rejuvenated” proteome 13, 21.

## Protein misfolding and aggregate formation

The process of protein folding is driven by the hydrophobic effect, where hydrophobic regions of the polypeptide chain are buried on the inside creating a specific three‐dimensional structure referred to as the native form 2, 22, 23. If the native form of a protein is disrupted and hydrophobic regions of the polypeptide exposed, the protein becomes misfolded and dysfunctional, and can accumulate in high molecular weight aggregates 10, 24. Mutations along with translational and transcriptional errors are intrinsic factors that promote protein misfolding and aggregation 25. Some proteins, rich in glutamine and asparagine residues, form highly organized aggregates called amyloids, whereas other proteins form less structured, disordered aggregates, commonly referred to as amorphous 26. Amyloid aggregates are characterized by tightly packed beta sheets and are, similar to non‐amyloidogenic amorphous aggregates, associated with neurodegenerative diseases 10, 27, 28.

External factors triggering protein misfolding include changes in temperature, oxidative stress, and specific drugs and chemicals 29, 30. Changes in temperature can disrupt hydrogen bonds and non‐polar hydrophobic interactions between amino acids in the polypeptide chain, thereby weakening the structure of the protein. The pH in the cell affects the protonation state of the protein, which is critical for non‐covalent interactions, and fluctuations in pH can therefore also destabilize the native form of a protein 31. Oxidative stress is mediated by reactive oxygen species (ROS) that can cause amino acid oxidation and/or cleavage of the polypeptide chain 32. Oxidative modifications by ROS can be reversible and important for protein function, but can also disrupt the structure of the protein leading to misfolding and aggregation 33. Protein carbonylation is a type of irreversible oxidation that results in a reactive carbonyl group (aldehyde or ketone). Protein carbonylation often leads to disrupted protein function and aggregation and has been widely used as a marker for oxidative stress and aging 32, 34, 35, 36.

## Aggregate recognition by the PQC system

Several classes of chaperones are involved in the recognition and elimination of protein aggregates. Small heat shock proteins (sHsps), referred to as holdases or aggregases, bind misfolded proteins and concentrate them in stable aggregates to prevent further interactions 37. Co‐chaperones of the J‐protein family (Hsp40) are also early participants in the aggregate recognition process: They bind unfolded regions of proteins as well as recruit and stimulate the ATPase activity of heat shock protein 70 (Hsp70). Hsp70 chaperones have protein‐folding capacities (foldases), are important for disaggregation, and aid in protein degradation by the ubiquitin proteasome system (UPS) 2, 24. Disaggregation does in some organisms also involve Hsp100 proteins such as the yeast Hsp104, which requires the assistance of Hsp70 and Hsp40.

**Figure 1:**
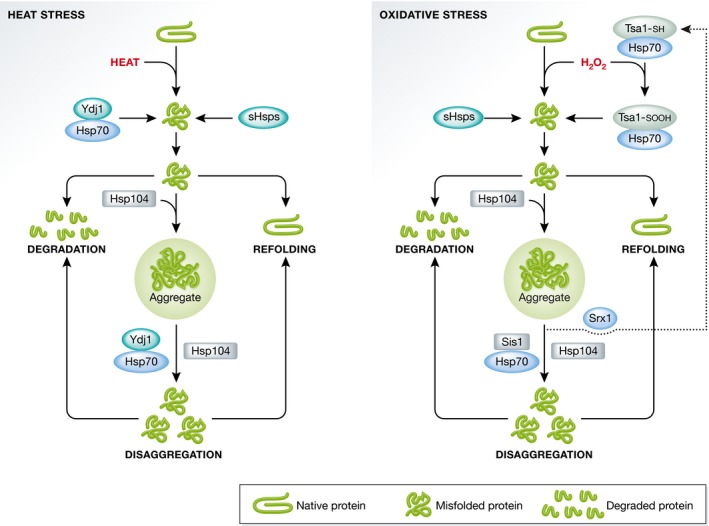
Differential PQC strategies during heat shock and oxidative stress In yeast, sHsps, Hsp40/Hsp70, and Hsp104 are sequentially recruited to denatured and misfolded proteins as well as to protein aggregates. The misfolded proteins can become refolded, degraded, or aggregated. Heat‐induced aggregates are disaggregated by the action of Hsp70 and Hsp104 together with the Hsp40 protein Ydj1, which binds to aggregates and enables refolding or degradation of the extracted misfolded protein species (left). Upon H_2_O_2_ exposure, Tsa1 is sulfinylated and recruited to misfolded proteins and protein aggregates along with Hsp70 and Hsp104 (right). Proteins misfolded upon oxidative stress can be degraded or refolded in a manner supposedly similar to the pathway for heat‐denatured proteins. However, disaggregation of H_2_O_2_‐induced aggregates depends on the core Hsp70/Hsp104 machinery together with the Hsp40 Sis1, while the heat‐related Ydj1 is not required. Furthermore, disaggregation upon oxidative stress is facilitated by Srx1‐dependent reduction in sulfinylated Tsa1. As for heat stress, these disaggregated proteins can either be refolded or degraded.

Interestingly, the specific set of chaperones recruited to a protein aggregate is dependent on the type of proteotoxic stress. In yeast, heat‐induced aggregates are recognized by the cooperation of sHsps (Hsp26/42), Hsp40, Hsp70, and Hsp104 (Fig 1). Aggregates formed by hydrogen peroxide (H_2_O_2_) are similarly recognized by the sHsps, but in contrast to heat‐induced aggregates, the recruitment of Hsp70/Hsp104 requires the peroxiredoxin (Prx) Tsa1 38 (Fig 1). Prxs are peroxide scavengers that undergo a functional shift to chaperones upon increased intracellular levels of H_2_O_2_ 39. This shift is mediated by the sulfinylation of a peroxidatic cysteine on Tsa1 and is reversed by the sulfiredoxin Srx1 38. The major cytosolic Hsp40, Ydj1, is required for efficient resolution of aggregates formed under heat stress 40, but is dispensable for aggregate resolution during H_2_O_2_ stress 38 (Fig 1). This could be explained by the fact that some Hsp40 chaperones such as yeast Ydj1 and human Hdj2 are inactivated during oxidative stress, by oxidation of their cysteine‐rich zinc finger domains 41. Clearance of peroxide‐induced aggregates is instead aided by Srx1‐dependent reduction in sulfinylated Tsa1, and the essential Hsp40, Sis1, which lack the oxidation prone zinc finger domain 38. Although Tsa1 localizes also to heat‐induced aggregates, it is not required for the formation or elimination of these types of aggregates, establishing Tsa1/Srx1 as PQC factors required specifically under oxidative stress.

The different chaperone requirements during heat and peroxide stress indicate that the contents or structure of aggregates formed during these conditions might be different. The distinct dynamics of aggregate movements observed under heat stress and H_2_O_2_ exposure support this notion; Tsa1‐associated aggregates formed under heat stress are mostly stationary, whereas the aggregates in H_2_O_2_‐treated cells are significantly more mobile 38.

## Distinct cellular sites for quality control

To study the process of aggregation in yeast and other systems, researchers have used different types of fluorescent misfolding substrates (Table 1). Upon heat stress and/or impairment of the PQC system, the temperature sensitive allele of the SUMO‐conjugating enzyme Ubc9 (Ubc9^ts^), the unassembled von Hippel–Lindau tumor suppressor (VHL), and the thermo labile luciferase all misfold and accumulate at multiple sites, called stress foci, CytoQs, or Q‐bodies, throughout the cytosol and at the surface of different organelles, including the endoplasmic reticulum (ER), mitochondria, and vacuole 16, 17, 42, 43, 44. Based mainly on microscopy, it is assumed that the fluorescent substrates used accumulate at stress foci due to their aggregation. The initial stress foci formation is energy‐independent but requires an intact ER 42, 44, suggesting that it is not a fully random event.

**Table 1:** Misfolding substrates used to study spatial PQC

	JUNQ/INQ	IPOD	References
VHL	x	x	16 17 42 43 47 57 72
Ubc9^ts^	x	x	16 17 42 43 44 45 46 47 57
Luciferase	x	x	17 42 43
Act1‐E364K	x	x	16
Rnq1		x	16 17 42 45
Ure2		x	16
Sup35		x	15
Htt103Q		x	16 70
Htt97Q		x	42
Gnd1^ts^	x	x	43
ΔssCPY*	x	x	38 43 52
GFP‐DegAB	x	x	51
Guk1‐7	x	x	129

Localization of studied aggregation substrates to known quality control sites.

**Figure 2:**
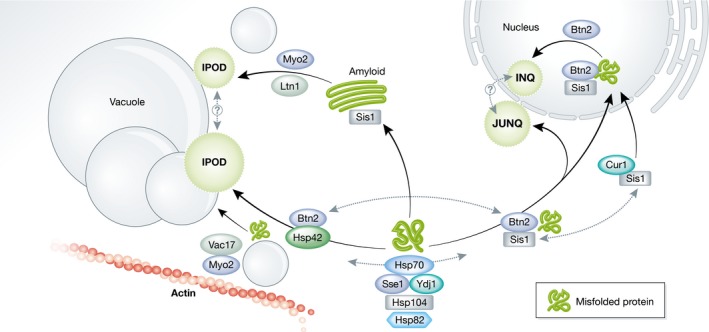
Sorting factors involved in the sequestration of protein aggregates toward quality control sites Misfolded and aggregated proteins are recognized by the yeast chaperone machinery: Hsp70s, Hsp40s (Ydj1 & Sis1), the Hsp90 Hsp82, and the disaggregase Hsp104. These chaperones are important for sequestering aggregated proteins into quality control sites: the juxtanuclear JUNQ, intranuclear INQ, and the perivacuolar IPOD sites. Aggregates bound by Btn2 and Sis1 are distributed toward JUNQ and/or INQ. The availability of Sis1 in the cytosol is regulated through competitive binding by Cur1, which translocates a fraction of the cytosolic Sis1 pool into the nucleus, making it unavailable for Btn2 binding. Inside the nucleus Btn2 acts as an aggregase, directing aggregated proteins into the intranuclear INQ inclusion. Btn2 also interacts with the small heat shock protein Hsp42 that is necessary for binding and targeting aggregates to the IPOD. Inclusion formation requires the actin cytoskeleton and vesicle trafficking, which is regulated by the vacuolar adaptor Vac17 and the motor protein Myo2. Amyloidogenic proteins, such as Htt103QP, are sequestered to the IPOD in a pathway independent of Hsp42 and require Sis1 and the E3 ligase Ltn1. This pathway also depends on Myo2‐associated vesicle trafficking, but does not require Vac17. The IPOD formed by amyloids might therefore be distinct from that formed by amorphous aggregates; however, the formation of amyloid IPODs has been shown to influence the formation and dynamics of amorphous inclusions in an up till now undefined manner. See text for more details.

Upon prolonged proteostasis stress, aggregates in stress foci coalesce, by an energy‐dependent process, into larger structures often referred to as inclusions 16, 42. It is not clear whether misfolded and aggregated proteins in stress foci and inclusions display structural differences, but the large inclusions display specific cellular localization: They are located to at least two distinct spatial quality control sites (sometimes referred to as compartments 16): the juxtanuclear quality control site (JUNQ) and the peripheral, vacuole‐associated insoluble protein deposit (IPOD) 16 (Fig 2). While heat‐denatured proteins locate to both sites, amyloidogenic proteins such as yeast prions or the mutated version of Huntingtin exon 1 are primarily targeted to the peripheral IPOD 16, 17, 42. The inclusion at JUNQ is highly dynamic in the sense that its misfolded proteins are rapidly exchanging with the cytosol 16. This rapid turnover is not seen in the IPOD, which exhibits almost no dynamic exchange with the surrounding cytosol, and was therefore originally thought to harbor terminally insoluble proteins 16, 17.

The energy dependence of inclusion formation is explained by the fact that many PQC factors, for example, the major Hsp70s (Ssa1 and Ssa2) along with the nucleotide exchange factor Sse1 and the Hsp40 co‐chaperone Ydj1, need ATP for their function 42, 45, 46 (Fig 2). Inclusion formation in yeast also requires the disaggregase Hsp104 and its ATPase activity, through an unknown mechanism 47, 48. One explanation for such ATP dependence could be that Hsp104 acts indirectly by modulation of the actin cytoskeleton 49, which has been shown to be involved in proper formation of inclusions (see sections below). In addition, the stress‐induced Hsp90, Hsp82, is important for inclusion formation, whereas its constitutively expressed homologue Hsc82 is not 42.

In yeast, misfolded cytosolic protein aggregates are also observed inside the nuclear membrane and it was suggested that this quality control site should be labeled the intranuclear quality control site (INQ) 43. Moreover, the intranuclear site seems to occupy a specific location within the nucleus, in close proximity to the nucleolus 43, 45, 46. INQ formation is induced also during genotoxic stress, and several INQ‐specific proteins have been identified during this stress, including proteins for chromatin remodeling and cell cycle control 50. However, as the same model substrates have been used in defining both JUNQ and INQ in studies during proteotoxic stress, it is difficult to make a clear distinction between the two sites. Thus, we henceforth refer to them collectively as JUNQ/INQ.

JUNQ/INQ appears to form first upon stress, whereas the IPOD forms later and remains longer upon the relief of proteostatic stress 16, 45, 47. This observation, and the fact that JUNQ/INQ is a highly dynamic structure enriched for proteasomes (see section on spatial sorting factors below), could be an indication that the juxtanuclear site is the preferred control site for misfolded proteins, and that substrates are only directed toward the IPOD when JUNQ/INQ is overcrowded or overwhelmed 15. In this scenario, misfolded proteins that fail to be degraded at JUNQ/INQ could be re‐routed to IPOD, which would require some kind of trafficking between JUNQ/INQ and IPOD. Whether such trafficking occurs is presently unknown. Another possibility is that misfolded proteins are sorted into JUNQ/INQ and IPOD based on their structural and physical properties and that proteins are sorted to IPOD as a protective measure, in other words, to protect functional, or partially functional, misfolded proteins from degradation (see below).

Once the spatial PQC system of the yeast cell has redirected aggregated proteins to inclusions, the clearance of these inclusions requires the disaggregase activity of Hsp104 together with the activity of Hsp70s and co‐chaperones 17, 47 (Fig 1). The cytosolic Hsp40 Ydj1 is essential for the refolding of misfolded and aggregated substrates, whereas this process is independent of the related Hsp40 Sis1 42, 51. This relationship between the cytosolic Hsp40s is explained by a competitive binding of substrates, where binding by Ydj1 directs the substrate for cycles of refolding, whereas binding by Sis1 targets the substrate for degradation 52. The clearance of inclusions is independent of the sorting factor Hsp42 42, while the sorting factors Hsp82 and Sti1 affect both spatial targeting as well as degradation 16, 42 (see section below on spatial sorting factors). Clearance of inclusions also requires an intact cortical ER and a functional UPS system, along with proteins for required for deubiquitination 42, 46.

Although the perivacuolar IPOD site persists for a longer period after stress, this inclusion is eventually cleared upon returning cells to non‐stress conditions. This clearance was shown to be dependent on the disaggregase Hsp104, suggesting that proteins within IPOD are not terminally misfolded 17, 43.

It should be noted that spatial compartmentalization of protein aggregates is not a secondary strategy or “backup plan”, but is rather a complementary control strategy that acts in parallel with temporal quality control. While inclusion formation is not essential for degradation, it may facilitate refolding/degradation by increasing the proximity of chaperones and their substrates 42, 53. Furthermore, the sequestration of misfolded proteins into inclusions decreases the exposed surface area and limits potential toxic interactions that could interfere with cellular functions.

In addition, it has been suggested that inclusion formation is a way not only to sequester damage but also to protect the proteome during times of stress 54. Studies using rapid and severe heat stress in yeast (46°C 8 min) demonstrated that entire protein complexes could be segregated into aggregates, and yet their function completely restored upon stress relief 54. It is possible that during spatial quality control, when aggregates are merged into larger inclusions at specific sites, some proteins that were part of the initial aggregates/stress foci are extracted and returned to their normal function. The process of temporarily sequestering functional components is seen also during stress granule formation, where mRNA and ribosomes are aggregated to initiate a global stall of translation 55. Interestingly, stress granules are formed on preexisting protein aggregates, evidently linking the two sequestration pathways 56. However, the possible functional contribution of stress granule formation in aggregate inclusion formation and spatial quality control is yet to be determined.

## Spatial sorting factors and mechanisms of inclusion formation

As all of the non‐amyloid substrates of spatial PQC localize to both IPOD and JUNQ/INQ, it does not seem to be the protein as such that determines its PQC destination. Rather, it is more likely that the spatial sorting is a result of the degree of misfolding, aggregate state/structure, and the types of protein motifs that are exposed for recognition by certain sorting factors 17 (Table 2; Fig 2).

**Table 2:** Spatial sorting factors and chaperones involved in spatial PQC

	JUNQ/INQ	IPOD	Regulates inclusion formation	References
Hsp104	x	x	+++	16 17 42 43 44 45 47
Ssa1/2	x	x	+++	42 46 57
Sse1	nd	nd	+++	42
Ydj1	nd	nd	+++	42 45
Sti1	nd	nd	+ (JUNQ/INQ)	16 43
Sis1	x	x	++ (JUNQ/INQ)	57 72
Hsp42		x	+++ (IPOD)	17 42 43 57 72
Hsp82	nd	nd	++	42
Mca1	x	x	−	45 72
Btn2	x	x	+++ (JUNQ/INQ)	43 57 72
Cur1	x		+	57
Vac17	nd	nd	++	63
Hsp26	x	x	−	17 42
Tsa1	x	x	−	38
Ltn1		x	+++ (IPOD)	69

Localization of studied sorting factors and chaperones to quality control sites and the dependence on their presence for proper inclusion formation. Major determinants of spatial sorting denoted with +++, less important factors ++, and factors displaying only a minor role in sorting are denoted with +. Chaperones and aggregate‐associated factors that do not affect spatial sorting are denoted with −. nd, localization in respect to quality control sites has not been determined.

Based on the enrichment of proteasomes at JUNQ/INQ and the rapid exchange between JUNQ/INQ and its surroundings 16, it was suggested that localization of misfolded proteins to this site could be linked to recognition by the UPS; that is, the ubiquitination status of a protein might act as a potential sorting signal. Initial studies showed that blocking ubiquitination decreased the ratio of misfolded proteins that were targeted to JUNQ, whereas addition of an ubiquitin tag to the IPOD‐exclusive substrate Rnq1 could partially redirect it to JUNQ 16. In line with this, ubiquitination was proven essential for the sequestration of proteasomal substrates in cells with limited chaperone function 51. While these data pointed toward protein ubiquitination as a possible sorting signal, later studies failed to demonstrate site‐specific immunostaining of ubiquitin, and showed that the misfolded substrate Gnd1^ts^ still localizes to both JUNQ and IPOD sites even when two major E3 ubiquitin ligases are deleted 43. Thus, while ubiquitination could be important in certain cellular contexts 51, there might be additional features of the misfolded proteins that enable their interaction with certain sorting factors and direct them to discrete quality control sites.

The small heat shock protein Hsp42 has been identified as a major sorting factor in yeast and is involved exclusively in sorting aggregated proteins into an inclusion at the peripheral IPOD. Consequently, deletion of this gene causes the majority of misfolded proteins to be redirected toward the JUNQ/INQ 17, 43, 57. The prion‐like N‐terminal domain of Hsp42 is essential for its localization to aggregates and the formation of IPOD 17. As deletion of Hsp42 does not completely abolish IPOD formation, there may be additional sorting factors involved in the formation of this quality control site, and it also remains to be elucidated whether the IPOD formed by amorphous aggregates corresponds to a different inclusion site than that formed by amyloids. In addition, the exact localization of the IPOD needs further clarification. The IPOD is localized to the vacuolar membrane, close to the pre‐autophagosomal structure (PAS) 15, 16, 47; however, the process of autophagy is not needed for IPOD formation or its dissociation 16, 42, 58. This could indicate that IPOD formation is restricted to a certain site at the vacuolar membrane, directed, and tethered by factors that are yet to be identified (see below).

For sequestration of proteins toward the nuclear control sites, the v‐SNARE binding protein Btn2, and to a lesser extent its paralog Cur1, have been implicated to function as sorting factors 57 (Fig 2). The Hsp40 co‐chaperone Sis1 and Hsp42 compete for binding to Btn2, where Btn2 either directs Sis1 to the nucleus or accompanies Hsp42 toward the IPOD site 50, 57. When in the nucleus, Btn2 functions as an aggregase, promoting coalescence of misfolded proteins into an inclusion 43. Cur1 appears located exclusively to the nuclear inclusion site together with Sis1 and could regulate the balance of sorting between JUNQ/INQ and IPOD by affecting the amount of Sis1 available in the cytosol for interaction with Btn2 57. In agreement with this, overproduction of Cur1 redirects cytosolic Sis1 to the nucleus and causes misfolded proteins to be partially diverted to the cell periphery 57, and limiting cellular levels of Sis1 results in a similar phenotype 43. Further, supporting the importance of available cytosolic Sis1 in spatial sorting of PQC substrates, co‐expression of prions or amyloid‐forming proteins have been demonstrated to sequester Sis1 from the cytosol and thereby inhibiting import of cytosolic substrates into the nucleus for their degradation 52. The role of Sis1 in spatial quality control might, however, be partly indirect as it also affects the load of misfolded proteins by regulating ubiquitination and degradation 51, 59.

As outlined above, JUNQ/INQ formation during proteotoxic stress requires Btn2 but is independent of Hsp42. However, during genotoxic stress intranuclear site formation depends on both Btn2 and Hsp42 50. As Hsp42‐dependent protein sorting has only been investigated in the context of IPOD formation during proteotoxic stress, the mechanism underlying its involvement in INQ formation during genotoxic stress is unclear.

Another protein associated with the formation of JUNQ/INQ is the Hsp90 co‐chaperone Sti1. Although not essential for JUNQ/INQ formation, deletion of *STI1* dramatically impedes the transport of misfolded proteins into nuclear deposits and multiple cytosolic aggregates are formed 16, 43. However, JUNQ/INQ formation is not affected when *STI1* is deleted in an *hsp42*Δ background, where the majority of misfolded proteins are routed to the nuclear inclusion 43. Thus, the defect in spatial quality control observed in Sti1‐deficient cells might be indirect and linked to an elevation in the levels of misfolded and ubiquitinated proteins in such cells as Sti1 regulates the ATPase activity of both Hsp70s and Hsp90s 60, 61. Sti1 has also been implied in the formation of peripheral inclusions of amyloidogenic proteins, but whether this is due to a direct or indirect function remains to be elucidated 62.

Some misfolded proteins require a functional actin cytoskeleton and/or transport along the cortical ER to reach their quality control site in addition to appropriate sorting factors 16, 17, 42. Furthermore, the Hsp104 disaggregase interacts with components of the endosomal trafficking pathway, and this association is even more pronounced upon heat treatment 63. Additionally, the deletion of genes required for endosomal vesicle trafficking and vesicle fusion to the vacuole severely impaired inclusion formation, whereas overexpression of proteins that accelerated vesicle movement also accelerated inclusion formation 45. These data highlight the involvement of vesicle trafficking and inter‐organelle communication in spatial PQC 42, 44, 47, 63, and based on these reports, the dependence on the actin cytoskeleton for IPOD formation might be the result of hetero‐ and homotypic fusion to the vacuole, which requires actin and actin‐remodeling proteins 64.

Interestingly, the deposition of beta‐sheet containing amyloids into inclusions seems to involve a somewhat different set of sorting factors and mechanisms. Amyloid‐forming proteins skip the intermediate step of stress foci formation and directly form a peripheral IPOD‐like inclusion 42, and in contrast to inclusion formation of amorphous aggregates, the sequestration route for amyloids is independent of Hsp42 17, 42 (Fig 2). Inclusion formation of amyloids requires the cooperation of Hsp70 together with the Hsp40 co‐chaperone Sis1 52, 62, 65 and is also aided by interactions with proteins rich in glutamine and asparagine 66, 67, 68. A genome‐wide screen also identified several components of the ribosome quality control complex (RQC), including the E3 ubiquitin ligase Ltn1, as being essential for inclusion formation of the amyloidogenic Huntington disease protein Htt103QP 69 (Fig 2). Similar to amorphous aggregates, Htt103QP amyloids interact with the actin cytoskeleton 69, 70, and mutations that reduce actin cable formation and/or actin integrity renders the Htt103QP cytotoxic 71.

Myo2‐mediated vesicle transport has been implicated in the inclusion formation of both amorphous aggregates and amyloids 58. However, the sequestration of amorphous aggregates depends on Myo2 interacting with the vacuolar adaptor protein Vac17, whereas Vac17 is not required for the spatial control of amyloidogenic proteins 63. Instead, it was suggested that amyloids might be delivered to the perivacuolar control site via vesicles along the cytoplasm‐to‐vacuole targeting (CVT) pathway 58. However, a direct interaction between aggregated proteins and functional vesicles has not been demonstrated.

Furthermore, protein aggregates were shown to localize in the proximity of lipid droplets, and the sterol‐composition of lipid droplets regulates the rate of inclusion clearance 72. It is therefore possible that sterols could act as chemical chaperones, aiding in dissolving and clearing aggregate inclusions. Another possibility is that fusion of lipid droplets to the vacuolar membrane facilitates import of IPOD‐residing misfolded proteins into the vacuole, such that they can be degraded by vacuolar proteases. However, further studies are required to investigate the exact involvement of quality control sites and spatial PQC in damage removal.

## Protein aggregation during cellular aging

Protein aggregates accumulate with time in several model organisms and are considered a hallmark of aging. This age‐related accumulation of damaged proteins may be a combined effect of increased levels of damaging agents together with a decreased capacity of the PQC system to remove damage. Supporting this is the fact that ROS levels and oxidatively damaged proteins increase with age in many organisms 73, 74. Furthermore, the capacity of the UPS and vacuole‐mediated autophagy declines with age leading to an age‐associated decay in PQC 75, 76. The connection between PQC, protein damage, and longevity is demonstrated by studies showing that deletion of major chaperones, ROS scavengers, or proteasome subunits leads to an increased load of non‐native proteins and protein aggregates, and reduces replicative life span in yeast 46, 77, 78, 79. Conversely, increasing the activity of PQC components mitigates the accumulation of protein aggregates in aged cells and extends life span 38, 45, 77, 79.

As many studies of protein aggregation have been performed using various immediate stresses as the cause of aggregation, much of the features of protein aggregates formed progressively through cell generations upon replicative aging remain elusive. There is however data demonstrating that age‐associated aggregates engage some of the same PQC proteins as stress‐induced aggregates. Peroxiredoxin Tsa1 is for example required for recognition of aggregates in aged yeast cells in a manner similar to aggregates formed under H_2_O_2_ exposure, and elevated levels of Tsa1 counteract accumulation of age‐induced protein aggregation and extend life span 38. Together with the fact that ROS and oxidatively damaged proteins increase with age it is reasonable to assume that H_2_O_2_ is one source of age‐associated protein aggregation. In addition, factors, such as the yeast metacaspase, Mca1, needed for resolution of heat‐induced aggregates have proven important also for longevity and PQC in aged cells 45, 46, 63. Moreover, analysis of the dynamics of aggregate movement suggests that aged cells harbor at least two distinct populations of aggregates; one being more stationary, resembling heat‐induced aggregates, and one being highly dynamic, similar to aggregates in H_2_O_2_‐treated cells 38. These findings suggest that age‐induced aggregation may follow routes similar to those triggered by both heat‐denaturation and protein oxidation.

Little is known about the protein content of aggregates in replicatively old cells, but it is likely that the many aggregation‐prone proteins are common for stressed and senescent cells. Newly synthesized, ribosome‐associated proteins, DNA‐binding proteins and proteins involved in translation are enriched in aggregates induced by heat, H_2_O_2_ and arsenite‐stress 54, 80. Aggregation‐prone proteins that are common for the different types of stressors showed a significant overlap when compared to proteins that aggregated during post‐mitotic, chronological aging in yeast 80, 81, and also show extensive overlap with proteins found to accumulate in age‐induced aggregates in the nematode *Caenorhabditis elegans* 82, 83, 84. Based on these data it appears that aggregate content is to most part not stress‐specific, and a distinct part of the proteome is particularly aggregation‐prone regardless of the proteotoxic stress. Thus, it is likely that aggregates formed during stress and aging contain similar protein species.

## Spatial PQC in aging cells

The aggregated proteins that accumulate in aging cells are sequestered into inclusions, however the similarities between these inclusions and the quality control sites that are formed upon stress‐induced protein aggregation are still unclear. Cells in early stages of aging are found with an Hsp70‐containing juxta/intranuclear inclusion, similar to the stress‐induced JUNQ/INQ 46. However, age‐induced inclusions are devoid of the JUNQ/INQ factor Btn2 and have also been shown to colocalize with the IPOD‐residing Rnq1 45, 85. A recent study suggested that age‐induced inclusions are neither JUNQ nor IPOD, and that they instead represent another distinct form of quality control site 85. It should be noted that the definitions of the quality control sites are ambiguous, especially for the peripheral IPOD inclusion: Initial studies used amyloids as IPOD markers, but later findings, as mentioned above, revealed that these substrates are sequestrated to a peripheral site by a specific, Hsp42‐independent route, different from that exploited by amorphous aggregates 17. Thus, it is not clear that the peripheral inclusion formed by amyloids and by non‐amyloidogenic proteins corresponds to the exact same site of quality control. It might be that when amyloids are expressed in cellular systems to function as an IPOD marker, the presence of these aggregate structures affects the routing of stress‐induced protein aggregates. In line with this, it was observed that the dynamics of aggregate formation and fusion was altered in the presence of the amyloidogenic HttQ97 42. Another study also showed that induction of Rnq1 expression in old cells already harboring inclusions, caused the disaggregase Hsp104 (and possibly other factors) to relocate from the existing inclusion toward the newly formed amyloid‐inclusion 85. These data indicate that there could be at least two different types of IPODs/peripheral inclusions: one being Hsp42‐dependent and one being Hsp42‐independent, and that the presence of either affects the formation and regulation of the other (Fig 2).

Deletion of Hsp42 reduces the fraction of aged cells displaying visible aggregate inclusions 85. As deletion of Hsp42 results in no obvious fitness defect and even extends replicative life span 17, 85, this could be interpreted as inclusion formation not being important for cytoprotection during cellular senescence. Additionally, or alternatively, it is possible that in *hsp42*Δ cells*,* misfolded proteins and their oligomers are rerouted toward JUNQ/INQ, which could promote and accelerate their degradation, leading to less accumulation in inclusions and an extended life span.

**Figure 3:**
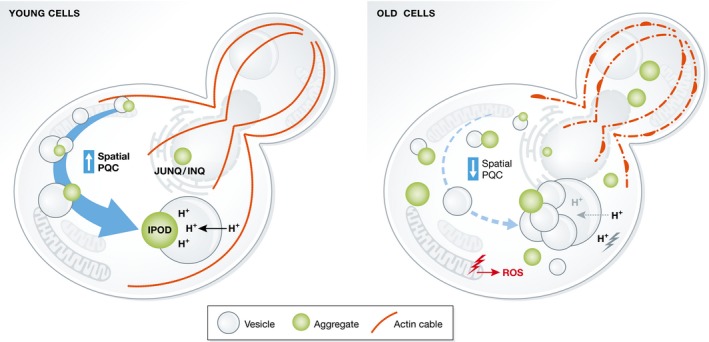
The loss of spatial PQC in old cells may be caused by the collapse of multiple cellular functions A cell's ability to sequester damaged and aggregated proteins into inclusions declines with age. This loss of spatial PQC could be due to the age‐dependent collapse in multiple functions. Inclusion formation is dependent on an intact actin cytoskeleton, the structure of which is lost during aging such that actin cables are less stable and F‐actin starts to accumulate in large aggregates. Dysfunctional mitochondria accelerate ROS production, causing oxidative modification of proteins and chaperones, thereby triggering increased aggregation and a decline in PQC. The loss of mitochondrial dysfunction is preceded by a loss of vacuolar pH control, which could lead to an acidification of the cytosol, inducing protein misfolding and aggregation. Loss of vacuolar pH control also decreases vesicle trafficking and vesicle fusion to the vacuole, a process that is important for inclusion formation.

While the exact nature of age‐induced inclusions remains unclear, a cell's ability to form these inclusions declines with increasing age 46, 69. The reason for this age‐dependent deterioration of spatial PQC is not known but may be caused by a simultaneous collapse of multiple systems 86 (Fig 3). The burden of protein damage increases with age, as a consequence of increased ROS produced by dysfunctional mitochondria, and a decline in UPS degradation 75, 87. It has also been shown that chaperones are especially prone to oxidation by ROS 36, 88. Thus, the increased load of aggregated proteins and the titration and inactivation of cellular chaperones could provide one explanation for the age‐associated loss of spatial PQC 51.

Reduced efficiency in the PQC upon aging could be linked also to the functional state of organelles. A loss of vacuolar pH control has been identified as one of the early events during aging in yeast, and is the result of fewer protons being transported into the vacuole 89. This defective pH control ultimately induces mitochondrial dysfunction and a reduced mitochondrial membrane potential, which have been shown to cause an increase in ROS production 89, 90, 91. As fewer protons are transported into the vacuole, the proton concentration in the cytosol might be elevated which could affect protein stability. Thus, the age‐related acidification of the cytosol could overwhelm the PQC system through elevated oxidation and protonation, ultimately causing a collapse of the spatial PQC. The loss of vacuolar acidification could also cause a breakdown in vesicle trafficking and fusion to the vacuole 92, 93, processes that are important for spatial sequestration of aggregated proteins 58, 63 (Fig 3).

The actin cytoskeleton is also affected during aging, with cells displaying a decrease in actin turnover and an accumulation of aggregated F‐actin 94, 95. The disruption of a functional actin cytoskeleton affects vesicle trafficking and fusion, mitochondrial inheritance, increases ROS accumulation and could also explain the inefficient inclusion formation observed in aging cells.

## Asymmetric inheritance of age‐accumulated protein damage

During yeast cell division, protein damage is to a large extent retained within the aged mother cell to produce a young and immaculate daughter cell, born with a full replicative potential 96, 97, 98, 99, 100. The process of damage segregation is an active and factor‐dependent process 45, 63, 70, 101. Disrupting this segregation by deletion of asymmetry‐generating genes (AGGs, see below) has a direct consequence on cellular life span, pinpointing accumulated protein aggregates as aging factors 45, 63, 77, 102.

Spatial sequestration of protein aggregates into the quality control sites (IPOD and JUNQ/INQ) within the mother cell restricts their inheritance 47, 103, and sorting factors for spatial PQC such as Hsp70s, Hsp40s and Hsp104 are thus indispensible for proper damage retention. Although there are conflicting reports regarding the dependency of the actin cytoskeleton for inclusion formation 17, 42, 47, 104, an intact actin cytoskeleton and cytoskeleton remodeling proteins are required for the selective retention of these inclusions in mother cells during cytokinesis 49, 77, 101, 102. Aggregated proteins are associated with actin cables, and the retrograde flow of actin, mediated by actin nucleation at the polarisome, prevents aggregates from entering the bud, and possibly also aids in inclusion formation 16, 43, 49, 77. The histone deacetylase Sir2 has also been implicated as an important factor for aggregate segregation, and affects this process possibly by regulating actin assembly via chaperonin containing TCP‐1 (CCT) 77, 102.

**Figure 4:**
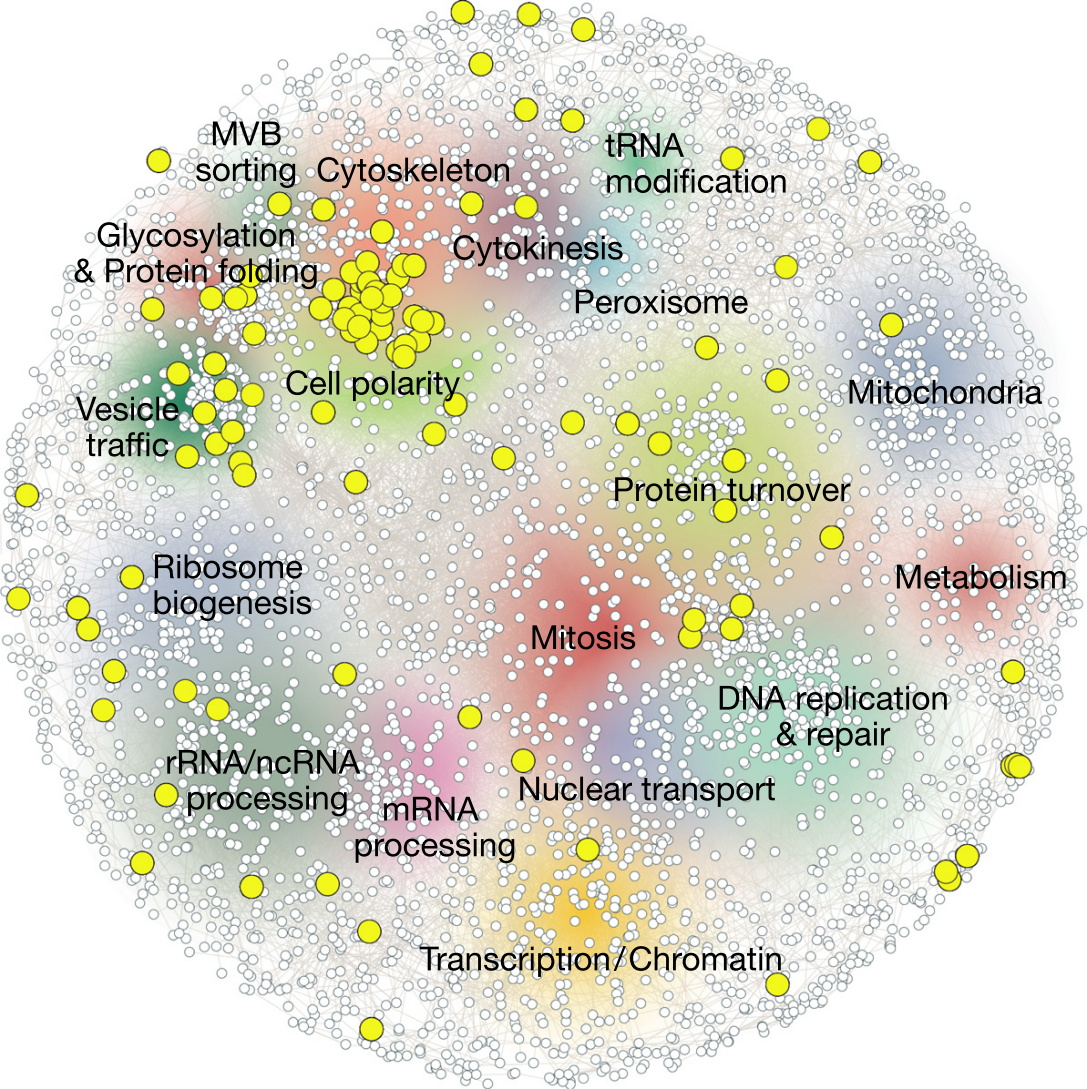
Asymmetry‐generating genes (AGGs) A map of the genetic landscape of a yeast cell, with genes required for the asymmetric inheritance of damaged and aggregated proteins denoted in yellow. Multiple cellular pathways are important for the generation of damage symmetry, but identified AGGs specifically cluster around functions related to protein folding, cytoskeleton organization, cell polarity, and vesicle trafficking, indicating that these cellular functions are major determinants of temporal and spatial quality control. Figure created using http://thecellmap.org. AGGs presented in this figure are also listed in Table 3.

The exact mechanism of how aggregates are linked to the actin cytoskeleton is not clear and studies have demonstrated close localization also between protein aggregates and the ER, mitochondria, vacuole and endomembrane vesicles 42, 44, 47, 63. These observations provide the possibility that the aggregate‐actin association could be mediated by aggregates being tethered to actin‐interacting organelles or vesicles. A recent genome‐wide screen aiming to identify factors required for generating damage asymmetry confirmed the importance of the actin cytoskeleton in this process 63 (Fig 4; Table 3). Additionally, this study identified several AGGs related to functions in vesicle transport and vacuolar functions and showed that AGGs are enriched in functions related to endomembrane trafficking, cytoskeleton organization and cell polarity (Fig 4; Table 3) 63. Deletion of these genes affects vesicle recruitment to actin cables, vesicle transport, and vesicle‐vacuolar fusion. Mutations in genes involved in these processes also impede inclusion formation and the retention of protein aggregates during cell division 63. On the other hand, overexpression of one of these genes, the vacuolar adaptor protein Vac17, efficiently boosted vesicle trafficking, inclusion formation, and damage asymmetry. This improved asymmetry was accompanied also by an extended replicative life span. The effect achieved by Vac17 overexpression was dependent on its binding to the actin‐interacting motor protein Myo2 63. Interestingly, Myo2 normally transports cellular material in an anterograde transport toward the bud 105, 106, and it remains to be elucidated how this motor protein affects the tethering of protein aggregates to actin cables while preventing them from entering the bud. One explanation could be that Myo2, by its interaction with Vac17 and Vac8 on vacuolar vesicles 106, brings aggregated proteins in close proximity to actin cables which impairs Myo2 motor function, allowing only passive movement of aggregates in the retrograde direction of actin cable growth. The observed results could also be explained by an indirect effect, where Vac17‐Myo2 act upstream to regulate vesicle trafficking and fusion, thereby affecting the retention of protein aggregates and their sequestration to the vacuolar membrane. The rejuvenation occurring during meiotic division of yeast, where all spores are cleared of damage 107, could also be linked to vacuole‐tethering of protein damage, as the entire vacuole is excluded from the cells during the sporulation process 108.

**Table 3:** Asymmetry‐generating genes (AGGs)

ORF	Gene name	GO biological function	References
YKL184W	SPE1	Biosynthesis	63
YIL122W	POG1	Cell cycle	63
YNL307C	MCK1	Cell cycle	63
YFL003C	MSH4	Cell cycle	63
YDR247W	VHS1	Cell cycle	63
YDL127W	PCL2	Cell polarity	63
YNL271C	BNI1	Cell polarity	102
YBR200W	BEM1	Cell polarity	102
YIL118W	RHO3	Cell polarity	102
YLL021W	SPA2	Cell polarity	49
YKR100C	SKG1	Cell wall organization	63
YDL035C	GPR1	Cellular response	63
YCR073C	SSK22	Cellular response	63
YCL026C	FRM2	Cellular response	63
YJR066W	TOR1	Cellular response	63
YNL305C	BXI1	Cellular response	63
YPL222W	FMP40	Cellular response	63
YHR206W	SKN7	Cellular response	63
YFL013C	IES1	Chromatin organization	63
YGL037C	PNC1	Chromatin organization	63
YCR082W	AHC2	Chromatin organization	63
YIL131C	FKH1	Chromatin organization	63
YDR254W	CHL4	Chromatin organization	63
YDR285W	ZIP1	Chromatin organization	63
YPL181W	CTI6	Chromatin organization	63
YDL042C	SIR2	Chromatin organization	96
YLR337C	VRP1	Cytoskeleton organization	63
YNL293W	MSB3	Cytoskeleton organization	63
YLR370C	ARC18	Cytoskeleton organization	63
YOR014W	RTS1	Cytoskeleton organization	63
YCR088W	ABP1	Cytoskeleton organization	63
YBL007C	SLA1	Cytoskeleton organization	63
YIL034C	CAP2	Cytoskeleton organization	63
YLR319C	BUD6	Cytoskeleton organization	63
YCR065W	HCM1	Cytoskeleton organization	63
YDR273W	DON1	Cytoskeleton organization	63
YBR109C	CMD1	Cytoskeleton organization	70
YDR356W	SPC110	Cytoskeleton organization	70
YDR208W	MSS4	Cytoskeleton organization	70
YLR212C	TUB4	Cytoskeleton organization	70
YLR319C	BUD6	Cytoskeleton organization	102
YCR009C	RVS161	Cytoskeleton organization	102
YFL039C	ACT1	Cytoskeleton organization	102
YMR032W	HOF1	Cytoskeleton organization	49
YCR092C	MSH3	DNA repair	63
YKR067W	GPT2	Lipid metabolism	63
YLR404W	SEI1	Lipid metabolism	63
YDR284C	DPP1	Lipid metabolism	63
YCL050C	APA1	Metabolism	63
YGR292W	MAL12	Metabolism	63
YGL062W	PYC1	Metabolism	63
YCR079W	PTC6	Mitochondrial process	63
YKL137W	CMC1	Mitochondrial process	63
YHR198C	AIM18	Mitochondrial process	63
YPL159C	PET20	Mitochondrial process	63
YCR083W	TRX3	Mitochondrial process	63
YDL119C	HEM25	Mitochondrial process	63
YLR091W	GEP5	Mitochondrial process	63
YDR256C	CTA1	Peroxisome	63
YBR082C	UBC4	Protein degradation	63
YMR119W	ASI1	Protein degradation	63
YFL007W	BLM10	Protein degradation	63
YOR197W	MCA1	Protein degradation	45
YDL126C	CDC48	Protein degradation	70
YPL240C	HSP82	Protein folding	63
YAL005C	SSA1	Protein folding	45
YLL024C	SSA2	Protein folding	45
YBL075C	SSA3	Protein folding	45
YDL229W	SSB1	Protein folding	45
YNL064C	YDJ1	Protein folding	45
YPL240C	HSP82	Protein folding	45
YJL034W	KAR2	Protein folding	70
YFL045C	SEC53	Protein folding	70
YLL026W	HSP104	Protein folding	77
YGR272C	YGR272C	Ribosome assembly	63
YGR276C	RNH70	Ribosome assembly	63
YOR293W	RPS10A	Ribosome assembly	63
YML063W	RPS1B	Ribosome assembly	63
YKR060W	UTP30	Ribosome assembly	63
YCR077C	PAT1	RNA processing	63
YCR063W	BUD31	RNA processing	63
YGR271W	SLH1	Translation	63
YNL014W	HEF3	Translation	63
YNL086W	SNN1	Transport	63
YCR069W	CPR4	Transport	63
YOR270C	VPH1	Transport	63
YCR073W‐A	SOL2	Transport	63
YNL326C	PFA3	Transport	63
YAL002W	VPS8	Transport	63
YCR067C	SED4	Transport	63
YEL005C	VAB2	Transport	63
YNL259C	ATX1	Transport	63
YGR254W	ENO1	Transport	63
YCL063W	VAC17	Transport	63
YKL175W	ZRT3	Transport	63
YCR068W	ATG15	Transport	63
YDL006W	PTC1	Transport	63
YOR106W	VAM3	Transport	63
YGR289C	MAL11	Transport	63
YER083C	GET2	Transport	63
YGL079W	KXD1	Transport	63
YIL085C	KTR7	Transport	63
YGL020C	GET1	Transport	63
YLR360W	VPS38	Transport	63
YCR075C	ERS1	Transport	63
YOR291W	YPK9	Transport	63
YAL014C	SYN8	Transport	63
YBR131W	CCZ1	Transport	63
YCR044C	PER1	Transport	63
YJR033C	RAV1	Transport	63
YPR149W	NCE102	Transport	63
YJL029C	VPS53	Transport	63
YGL084C	GUP1	Transport	63
YNL275W	BOR1	Transport	63
YOR132W	VPS17	Transport	63
YFR019W	FAB1	Transport	63
YEL013W	VAC8	Transport	63
YOR326W	MYO2	Transport	70
YBR080C	SEC18	Transport	70
YLR268W	SEC22	Transport	70

Names and annotations for genes depicted in Fig 4 as yellow nodes. Genes that are involved in the generation of damage asymmetry in yeast, where aggregated and misfolded proteins are retained in the mother cell during cell division to produce a rejuvenated progeny.

In addition to mechanisms that limit aggregate inheritance, cells have also evolved a process of aggregate removal so that any damage that accidentally leaks into the daughter cell is eliminated 45, 70. This removal is achieved either by disaggregation/degradation or by retrograde transport of aggregates back into the mother cell. As expected, most of the AGGs that have been studied affect both processes of aggregate retention and removal 45. Exceptions to this rule were the Hsp70 Ssa3 together with the metacaspase Mca1 that was found to solely regulate the process of aggregate removal. The specific role of Ssa3 in aggregate removal remains unknown, while the role of Mca1 is a combination of protease‐independent and protease‐dependent functions. Mca1 can act as a co‐chaperone and buffer against deficiencies in cytosolic Hsp40s and could possibly mediate its protease‐dependent effect by pre‐cleaving substrates and facilitating their subsequent removal by the proteasome 45, 109, 110.

## Spatial quality control in higher eukaryotes

Some features of inclusion formation and damage retention that are seen in yeast are conserved also in higher eukaryotes. An inclusion site, named the “aggresome”, accumulates close to the nucleus in mammalian cell systems and sequestration of damage to this site is essential for asymmetric inheritance and progeny rejuvenation 72, 111, 112, 113. Although functionally similar to the yeast JUNQ, aggresomes are enclosed by a vimentin cage and are formed in a dynein‐ and microtubule‐dependent manner, as opposed to the actin dependence suggested for the yeast protein deposits 17, 72, 112. Targeting of misfolded proteins to the aggresome includes both ubiquitin‐dependent and independent sorting signals 114, 115 and seems to share many of the sorting factors that have been identified in yeast 16, 72, 116, 117, 118.

Asymmetric distribution of cell fate determining factors is a process important for stem cell renewal and cell differentiation 119, 120. In addition to the cell fate determining factors, damaged proteins have been observed to segregate asymmetrically in stem cells during cytokinesis 72, 113, 121. Interestingly, the inheritance of protein damage in dividing stem cells is tightly linked to longevity and cell fate, where the cell destined for a long life is rejuvenated, whereas the cell with the shortest lifecycle inherits the damage 113. Germ line stem cells retain damage to give rise to damage‐free progeny that will outlive the mother, while for intestinal stem cells that are present throughout the organism's lifetime, the asymmetric distribution is the opposite 113. These long‐lived intestinal stem cells segregate damage to the differentiating progeny cells that are replaced approximately every week.

Involvement of vesicle trafficking in aggregation and cytotoxicity has also been reported for other eukaryotic systems than yeast. Mutations in proteins involved in endocytosis and actin function dramatically aggravate cytotoxicity of disease‐prone proteins such as Huntingtin and α‐synuclein, and these amyloid aggregates interact with components of the endocytic trafficking machinery 122, 123, 124, 125, 126. This interaction of aggregated proteins with the endocytic machinery could be a conserved mechanism of spatial sequestration. Thus, accumulation of misfolded disease proteins may obstruct accurate inclusion formation and instead lead to inhibition of endocytosis and toxicity 123, 127. The interaction with vesicles and the inhibition of their proper trafficking could be an explanation for why the toxicity of disease‐prone proteins is most pronounced in neuronal cells, as these cells rely on vesicle trafficking for appropriate synaptic function. Indeed, it has been shown that mutant Huntingtin associates with synaptic vesicles and inhibits neurotransmitter release 128.

## Concluding remarks

The existence and concept of spatial PQC is a rather recent addition to the many facets of proteostasis. The existence of such spatial control became manifest with data demonstrating that damaged and aggregated proteins can be spatially retained in one cell during mitotic division 77, 96 and that misfolded proteins are sequestered into specific cellular and organelle‐proximal depositions sites upon their aggregation 16. Experiments aimed at deciphering how spatial control is attained mechanistically suggest that spatial quality control requires many more factors in addition to those involved in temporal PQC. As reviewed herein, such factors include those involved in: (i) inter‐organelle communication by endomembrane trafficking; (ii) polarity establishment; (iii) formation and dynamics of actin cytoskeleton; and (iv) aggregate sorting by, for example, Hsp42, Btn2, and Ltn1. While the identification of these components and processes suggests that an almost bewildering arsenal of factors are required for proper control of protein damage, it pinpoints key cellular processes that may be alternative targets, beside canonical PQC factors such as chaperones, for boosting quality control in aging cells. Indeed, it raises the question if the increased protein aggregation, or aberrant aggregate formation, observed during aging is really due to a diminished activity of the canonical temporal PQC, as is often claimed, or due to reduced activity of auxiliary processes, such as endomembrane trafficking and inter‐organelle communication.

While pointing to new venues for approaching quality control in aging cells and organisms, many questions remain to be answered regarding the nature of spatial quality control. For example, is there exchange of substrates between, for example, IPOD and JUNQ/INQ, can there be only one IPOD or JUNQ/INQ in a cell, what is the nature of triage and targeting of misfolded substrates to these different quality sites, and are there new quality control sites to be discovered when expanding the misfolded substrates used and the conditions causing their misfolding?

It is also unclear to what extent the IPOD/JUNQ/INQ deposition sites formed during a heat shock (or other stresses) are similar to those observed in aging cells and whether the same factors are required in their formation. While protein misfolding and damage is generated in an instantaneous manner during a heat shock, such damage accumulates over generations during replicative aging, which might call for other routes and mechanisms of spatial quality control. Nevertheless, at least some factors required for retaining heat‐induced aggregates in mother cells are required also for the retention of aggregates formed upon aging 38, 45, 63, 77, 102.

As more data are generated on spatial quality control, a more stringent nomenclature concerning spatial PQC sites might be attainable. For example, since data have emerged demonstrating that the formation of vacuole‐proximal IPODs containing amyloid aggregates (e.g., Htt103QP and Rnq1) and amorphous aggregates (e.g., Ubc9^ts^ and VHL) require different factors for their formation, IPODs might need to be divided into “subfamilies” depending on the genetic determinants required to form them. A similar nomenclature based on cytological readouts, genetic determinants, substrate specificities, and specific stress (aging) conditions may in the future be achievable for other spatial quality control sites as well. Such amendments of definitions and nomenclature are only natural for a rather young subject matter such as spatial PQC and might help progress in this fascinating field of proteostasis research.

## Conflict of interest

The authors declare that they have no conflict of interest.

**Box 1: In need of answers**

- What are the additional genetic determinants required for the formation of the currently known quality control sites?
- How are substrates identified and targeted to inclusions at these different control sites?
- Is there any exchange of substrates between identified quality control sites?
- Are there more types of sites to be discovered when expanding on the misfolded substrates used and the conditions causing their misfolding?
- How do the control sites identified in stressed cells (e.g., heat shock) relate to those observed in aging cells with respect to location, content, and sorting factors?
- Is the accumulation of aggregated proteins upon aging a consequence of a decreased activity of the canonical PQC or the result of a reduced activity in spatial PQC?
